# COVID-19 at War: The Joint Forces Operation in Ukraine

**DOI:** 10.1017/dmp.2021.88

**Published:** 2021-03-25

**Authors:** John M. Quinn V, Trisha Jigar Dhabalia, Lada L. Roslycky, James M. Wilson V, Jan-Cedric Hansen, Olesya Hulchiy, Olga Golubovskaya, Mykola Buriachyk, Kondratiuk Vadim, Rostyslav Zauralskyy, Oleg Vyrva, Dmytro Stepanskyi, Pokhil Sergiy Ivanovitch, Alla Mironenko, Volodymyr Shportko, John E. McElligott

**Affiliations:** 1 Charles University, First Faculty of Medicine, Institute of Hygiene and Epidemiology, Prague Center for Global Health, Prague, Czech Republic; 2 Black Trident Defence and Security Consulting Group LLC, Sheridan, Wyoming, USA; 3 M2 Medical Intelligence, Inc., Reno, Nevada, USA; 4 StratAdviser Ltd., Paris, France; 5 P.L. Shupyk National Medical Academy of Postgraduate Education, Kyiv, Ukraine; 6 Bogomolets National Medical University, Kyiv, Ukraine; 7 Military Medical Clinical Center of North Region of Ukraine, Kharkiv, Ukraine; 8 Efferent Medicine Department, Kremenchuk Maternity Hospital, Kremenchuk, Poltava, Ukraine; 9 Bone Tumor Department, Ukrainian National Academy of Medical Sciences, Sytenko Institute of Spine and Joint Pathology, Kharkiv, Ukraine; 10 Department of Microbiology, Virology, Immunology and Epidemiology, Dnipro Medical Academy of the Ministry of Health of Ukraine, Dnipro, Ukraine; 11 Laboratory of New and Little-Explored Infections Disease, Mechnikov Institute of Microbiology and Immunology, National Academy of Medical Sciences of Ukraine, Kharkiv, Ukraine; 12 Department of Respiratory & Viral Infections, L.V. Gromashevsky Institute of Epidemiology & Infectious Diseases, National Academy of Medical Science of Ukraine, National Influenza Center, Kyiv, Ukraine; 13 Ukrainian Military Medical Academy, Kyiv, Ukraine; 14 Maricopa County Medical Society, Phoenix, Arizona, USA

**Keywords:** health security, war in Ukraine, COVID-19, NATO

## Abstract

The ongoing pandemic disaster of coronavirus erupted with the first confirmed cases in Wuhan, China, in December 2019, caused by the severe acute respiratory syndrome coronavirus 2 (SARS-CoV2) novel coronavirus, the disease referred to as coronavirus disease 2019, or COVID-19. The World Health Organization (WHO) confirmed the outbreak and determined it a global pandemic. The current pandemic has infected nearly 300 million people and killed over 3 million. The current COVID-19 pandemic is smashing every public health barrier, guardrail, and safety measure in underdeveloped and the most developed countries alike, with peaks and troughs across time. Greatly impacted are those regions experiencing conflict and war. Morbidity and mortality increase logarithmically for those communities at risk and that lack the ability to promote basic preventative measures. States around the globe struggle to unify responses, make gains on preparedness levels, identify and symptomatically treat positive cases, and labs across the globe frantically rollout various vaccines and effective surveillance and therapeutic mechanisms. The incidence and prevalence of COVID-19 may continue to increase globally as no unified disaster response is manifested and disinformation spreads. During this failure in response, virus variants are erupting at a dizzying pace. Ungoverned spaces where nonstate actors predominate and active war zones may become the next epicenter for COVID-19 fatality rates. As the incidence rates continue to rise, hospitals in North America and Europe exceed surge capacity, and immunity post infection struggles to be adequately described. The global threat in previously high-quality, robust infrastructure health-care systems in the most developed economies are failing the challenge posed by COVID-19; how will less-developed economies and those health-care infrastructures that are destroyed by war and conflict fare until adequate vaccine penetrance in these communities or adequate treatment are established? Ukraine and other states in the Black Sea Region are under threat and are exposed to armed Russian aggression against territorial sovereignty daily. Ukraine, where Russia has been waging war since 2014, faces this specific dual threat: disaster response to violence and a deadly infectious disease. To best serve biosurveillance, aid in pandemic disaster response, and bolster health security in Europe, across the North Atlantic Treaty Alliance (NATO) and Black Sea regions, increased NATO integration, across Ukraine’s disaster response structures within the Ministries of Health, Defense, and Interior must be reinforced and expanded to mitigate the COVID-19 disaster.

In November 2013, Ukraine’s democratically elected government decided to align closer with the European Union; economically, politically, and socially. In 2014, Russian special forces quickly seized multiple municipal buildings throughout Ukraine particularly in Ukraine’s illegally annexed Crimea, and its eastern region of Donbas (ie, Luhansk and Donetsk). Shortly thereafter, the Russian federal government, unilaterally and unsanctioned, annexed Crimea. Russian tanks invaded Donbas igniting the current armed conflict on Ukraine’s eastern flank. Social and political disruption spread through streets and cities. Families and economic prospects have been destroyed. Many warfighters and civilians are killed and wounded weekly.

Infectious diseases do not respect borders or other country barriers. Conversely, state sovereignty is threatened during times of pandemic crisis, exacerbating the disaster response likely to increase preventable morbidity and mortality. Russia has invaded and currently occupies eastern Ukraine and the Crimean Peninsula; roughly 1.8-5 million Ukrainian citizens are at increased health security risk from war, and now in addition from the threat of COVID-19. The Joint Forces Operation (JFO) in Ukraine evolved out of the Anti-Terrorist Operation (ATO) where defense forces are fighting Russian-backed proxies in Donbas.

Thus, Russia’s hybrid and multi-domain war against Ukraine illustrates the Russian foreign policy of strategic separatism. It results in “frozen conflicts” aimed at the reestablishment of Russian empirical hegemony. Examples span the post-Soviet space of Transnistria, Nagorno-Karabakh, South Ossetia, and Abkhazia. These frozen conflicts represent a hellscape of borderless security operations and dearth of health-related infrastructure where health-care staff are forced to migrate to areas of safety. Disaster response is further hindered as resources are allocated to warfighting efforts against an invading and occupying force.

The onset of the COVID-19 crisis has increased the potential of heightened tensions in Russia’s relations across the NATO alliance and complicated the prospects for cooperation, ceasefire, or an end to the war in Donbas. The conflict in Ukraine risks greater escalation if Russia expands its presence in Ukraine or affronts NATO countries directly due to the chaos brought on by failed responses and growing case counts. The possibility of additional incursions into Ukraine by Russian forces, or a sub-threshold Article 5 NATO security event may be closer than ever before.

A sovereign, independent, and stable Ukraine, committed to democracy, the rule of law, and in support of health security, will uphold Euro-Atlantic security and add value to the NATO alliance. Therefore, greater support of Ukraine’s Euro-Atlantic integration, standardization, and higher activity levels from the relevant institutions from its Euro-Atlantic partners are required.

## Aim

This study highlights the unique challenges arising from the presence of COVID-19 during active conflict in Europe. Said simply, interoperability and biosurveillance information sharing across agencies are needed now. The specific challenges faced by the Ukrainian Ministry of Defense, Ministry of Health, and Ministry of Internal Affairs are that they simultaneously struggle to fight invading Russian forces and establish effective public health responses to an increasing pandemic.

## Disease Prevention and Control

Infectious and communicable diseases thrive during wars, conflicts, and disasters. Ukraine is no different, but COVID-19 certainly is a new threat to health security in conflict zones. For instance, Ukraine suffered a polio outbreak, a completely preventable illness through vaccination, between 2014 and 2017. An additional measles outbreak, which began in 2016 and continues to the present day in Ukraine, has seen an increase from 78 to 3300 cases in the past 4 years. According to the WHO, Ukraine reported 56,802 cases of measles between January 1 and November 5, 2019.^1,2^

Tuberculosis (TB) persists throughout Ukraine, especially in vulnerable communities and among HIV patients. Although the TB vaccine Bacillus Calmette-Guérin (BCG vaccine) is widely used in many countries, its effectiveness in preventing TB is still a matter of clinical debate. The incidence of TB among the pediatric population is on the rise. In 2015, 826 children fell ill (of whom 33 were under 1 y old, and 379 under 4 y old), which is 8% more than in the previous period. In Ukraine, the estimated incidence of TB was 94.0 cases per 100 thousand people, but only 70.5 cases per 100 thousand people were found; roughly 25.0% of cases of TB patients are undetectable. Infectious disease is a major threat to health security in Ukraine and for poorly governed spaces, such as those in war, conflict, and disaster; COVID-19 makes this worse.

Vaccine-preventable disease, in the form of primary prevention, is the first barrier of defense for any community, population, or country. Measles, mumps, rubella, polio, diphtheria, tetanus, certain types of meningococcal meningitis, hepatitis A and B, among others, are just a sample of vaccine-preventable infectious diseases on a given country’s immunization schedule. COVID-19 vaccines, with varying efficacies against new varants, are available but it may take years before a viable option will provide widespread and defintive protection, if ever. Herd immunity is defined as those in a population that have seroconversion in their immune system to combat disease from natural infection or a vaccine; how long this immunity lasts for COVID-19, if at all, is not yet described in the evidence base currently available. Not all people who are vaccinated will seroconvert in their immune system and be immune to the disease, and some vulnerable and immunocompromised patients cannot receive the vaccine as it may be harmful to them based on their preexisting medical conditions. Herd immunity to COVID-19 may never occur, and if it does, for how long it will last may be too short to offer any level of protection for vulnerable communities. New variant strains pose a specific threat to health security.

Primary health-care services were destroyed during the Russian invasion; the Ukrainian pediatric population suffered most with reduced access to basic primary prevention.

Challenges to primary prevention explode when war and violence erupts and there is forced migration and people flow, displaced people, and a reduction or complete elimination of access to primary health-care sources to provide vaccination and immunization programs. In Ukraine, this has persisted throughout regions caught in the middle of war since 2014 and many people have not received adequate vaccinations and are vulnerable to preventable illness as evidenced by polio and measles outbreaks there. Increased operations with the Centers for Disease Control and Prevention (CDC), the Department of Defense, and NATO can mitigate these many growing health security threats for Ukraine, a sovereign nation at war with Russia.

## COVID-19

COVID-19, caused by severe acute respiratory syndrome coronavirus 2 (SARS-CoV-2), has no definitive treatment beyond supportive care. A vaccine remains elusive. The only primary prevention for COVID-19 consists of social distancing, aggressive handwashing, isolation and quarantine, limited movement and travel, and, most importantly, the protection of vulnerable potential patients. Understanding the natural history of disease and epidemiology of the disease is at its infancy for COVID-19. COVID-19 is efficiently transmitted by respiratory mechanisms. The pandemic threatens the health security and economic prosperity of millions of NATO state citizens in a way that much more limited terrorist strikes or the even that of potential Russian incursion on a NATO state do not.^3^ It is our belief that countries united in alliance to defend against a common enemy may perform better across partner institutions in terms of civil, united response to pandemics.

One of the major challenges facing public health researchers is determining the case fatality risk ratio of COVID-19. The case fatality ratio varies considerably from country to country and even between regions or cities within countries. In Ukraine, the case fatality rate, also referred to as “infection fatality rate” (as of January 2021) was estimated at 1.8%.^a^ COVID-19 mortality rates depend on the patients’ age, preinfection health, and the quality and capacity of the local health system to identify positive cases early and symptomatically treat aggressively. Fatality rates across countries differ widely and may not be understood for many years. The health-care system in occupied territories is poorly equipped to handle the management and spread of COVID-19 or to conduct the diligent testing and tracing of cases that is required to slow the transmission.^4^

Since the detection of the first COVID-19 case in Ukraine on March 3, the number of cases continues to rise daily and the risk of local transmission increases. A majority of projections put the end of 2020 and start of 2021 in Ukraine at anywhere from 10,000 to 20,000 cases a day. This was not observed, and a sharp decrease in cases were seen over the winter period and New Year, as low as 4 to 5500 cases/d. However, as of mid-January 2021, daily case counts grew well above 9000 a day. The new COVID-19 viral variants identified in the United Kingdom, South Africa, and Japan may cause a logarithmic increase as a worst-case scenario throughout Ukraine. The case counts that errupted in April 2021 hit the mark of over 19,000 cases in a 24 hour period and pushed total infections over 2 million and over 40,000 dead in Ukraine. Risk is particularly high in non–government-controlled areas with a large proportion of the population in their 6th decade of life and a decimated health-care system through the cumulative impact of years of armed conflict and neglect.^4^

However, effective disaster response measures by state health systems, especially those in active and open conflict, will likely contribute to lower mortality; Ukraine is no exception. Surveillance and testing policies, including milder cases and asymptomatic infections that may not be captured in countries that are restricting testing to severe or hospitalized patients, may play a role.^5^ South Korea reported a 1.3% case fatality ratio where a robust testing program was implemented, performing millions of tests since the onset of the pandemic.^6^ Anecdotally, broad access to accurate testing saves lives and may help to limit the spread of COVID-19; this is inherently difficult when clinical governance in war and occupied zones is challenged or laboratory infrastructure is strained or unavailable for vulnerable communities.

Based on evidence from late October 2020, the SARS-CoV-2 virus can stay in droplet form and infect other people in the air, as well as through sneezing, coughing, and other respiratory secretions and is labeled a potential airborne threat. Also, it is able to live on various surfaces for many hours and days. The COVID-19 (and its variants) transmission details continue being researched and the research outcomes will better contribute to the understanding about variants and transmissibility and even mortality. This disease profile offers a myriad of challenges for public health throughout the JFO. Living quarters for civilian and vulnerable populations currently in Luhansk and Donetsk, and at the border crossings of the contact line in Donbas, are squalid, have faced persistent hygienic challenges, and often require long wait times in poorly ventilated buses, non–temperature-controlled rest stops, and holding areas. This warzone environment may be the perfect area to spread COVID-19 if measures such as face masks, enforced social distancing, and access to accurate rapid testing are not supported.

## Ukraine Health-Care Infrastructure

From 2010 until 2013, Ukraine was subjected to a systematic gutting of defense, health, and disaster preparedness structures throughout its ministries. This helped pave the way for the Russian Federation to violently annex, invade, and occupy territories of Ukraine. Ukraine’s state sovereignty has been continuously challenged during the 3 decades of its independence. In an effort to reform under extreme duress, the acting Minister of Health (during 2016 to 2019) enacted a rash of mixed reforms to public health and disaster preparedness. The result was an unpopular reduction of epidemiologists in support of public health-care systems. This reform led to shortcomings in the polio outbreak, which began in 2015 and persisted through 2017, as well as the exacerbation of the persistent measles outbreak throughout Ukraine to 2021.

Within the current conditions of Ukrainian national health care, the country was ill prepared to rapidly identify the spread of COVID-19 and respond efficiently at the initial stages of the pandemic. This was partially due to insufficient logistical equipment of hospitals; the lack of qualified specialists on infectious diseases, virology, epidemiology; the arrangement of infectious departments throughout Ukraine’s regions; surveillance and appropriate testing labs and the lack of adequate personal protective equipment (PPE); enacting a policy of social distancing, among many other shortcomings in a strained health-care system. Much of this has been overcome through summer and fall of 2020, but the damage has already been done and implementation remains scattered into 2021 across clinical sites.

Additionally, during the winter period from 2020 to 2021, climate problems are identified and complicate COVID-19 detection and diagnosis. In particular, temperature and humidity were not specific throughout Ukraine (most of the time from 0 * C to + 5 + 10 * C). These conditions may contribute to the spread of infectious disease, and, therefore, caused an increase in seasonal incidence of various additional respiratory diseases, which exacerbated challenges for primary care services and doctors at all levels of care (pneumonias, seasonal influenza, etc). In January 2020, more than 70 cases of pneumonia, officially identified by doctors, were confirmed on the territory of Ukraine as complications from seasonal influenza, in particular, the Ministry identified Ternopil, Ivano-Frankivsk, and Volyn regions of concern. In some cases, the symptomatology will be considered by doctors to be a complication of influenza.

COVID-19 testing was not available at this time for many regional medical centers. To add to the challenges, COVID-19 testing was slow to start and inadequately carried out as there were limited reverse-transcriptase polymerase chain reaction (RT-PCR) capabilities in the country; or to the newer accurate rapid tests with poorly evidenced sensitivity and specificity. In addition, critical care infrastructure had a very low baseline to manage or be resilient to a significant patient surge, a requirement inadvanced critical care. At the same time, urgent measures nearby were taken in the Czech Republic, Italy, and Poland to counter the spread of COVID-19. As the number of patients increased, health security decreased. It was only after the first COVID-19 test kits began arriving in February 2020 that doctors fully began to have the capacity to confirm diagnosis of COVID-19. Although technology has advanced since this time, with broader access to PCR and antigen testing, no genomic sequencing technology devoted to the identification of variant strains exists as of April 2021.

## Ukraine Emergency Measures

The Ministry of Health, the National Security and Defense Council, and the President of Ukraine made urgent decisions in mid-March 2020. The March 13, 2020, National Security and Defense Council Decision On Urgent Measures to Guarantee National Security in the Event of an Outbreak of the Acute Respiratory Illness COVID-19 with SARS-CoV-2 coronavirus was put into effect by Presidential Decree No. 87/2020 dated March 13.^7^ The control from the presidential directive focused on limited travel and movement restrictions of foreign nationals and Ukrainian nationals who may enter or leave Ukraine.

The new law also provides for urgent and comprehensive analysis of the health-care system’s ability to respond effectively to the current coronavirus pandemic. This presidential directive also ensures the drafting and submitting to the Verkhovna Rada of Ukraine a bill on amendments to the Law of Ukraine On Public Procurement regarding the nonapplication of the effect of the aforementioned law to cases where the procurement pertains to medical supplies and specialized medical equipment needed to assist patients with COVID-19.

Under this impactful and comprehensive presidential plan, there is an additional acquisition of test kits for the detection of COVID-19 at checkpoints across the state borders of Ukraine and checkpoints of entry from temporarily occupied territories in Donetsk and Luhansk regions and Crimea, as well as, immediate financing of the production of COVID-19 RT-PCR diagnostic tests by the Institute of Molecular Biology and Genetics of the National Academy of Sciences of Ukraine.

One major challenge for all states responding to COVID-19 is exposure of first responders. This is of particular concern for countries in active war. The presidential directive also has a provision of individual means of protection to medical personnel, law enforcement and emergency services involved in anti-epidemic measures, as well as vulnerable contact with patients with COVID-19. The decree will stimulate the production of PPE and medical supplies by domestic enterprises and specialized medical equipment to assist patients with COVID-19. It also transfers government employees to staff 24/7 operations aimed at counteracting the spread of COVID-19. These steps will provide great support, and should be supported, where possible by Ukraine’s Allies; the challenges for a country at war are significant.

## Humanitarian Response

The United Nations (UN) has rolled out the current COVID-19 Emergency Response Plan (ERP), which distinguishes the financial risk for the eastern Ukraine and how it must be integrated to the overall country Ukraine Humanitarian Response Plan for 2020.^8^ This Plan focuses on addressing life-saving humanitarian needs and protection in response to the conflict, especially in light of the health security threat posed by COVID-19. The COVID-19 ERP is built upon and fully integrates the financial requirements outlined in the National Strategic Preparedness and Response Plan, developed by WHO and key partners.

The COVID-19 ERP extends the time frame for the financial risk to 9 month (the WHO-supported National Strategic Preparedness and Response Plan has a 3-month horizon) and also includes complementary assistance that is required, beyond the health dimension and support disaster response infrastructure. The UN/WHO Health Cluster process is vibrant in the disaster response for Ukraine.

The health cluster observed the Ministry of Health for Ukraine approve a vaccination strategy in Ukraine; this includes discussion about a national immunization strategy with defining phases and platforms for service delivery. Planned timelines to start COVID-19 vaccination from March 2021 to March 2022 vaccination (4 phases according to targeted groups will be done during the mentioned timeline) and vaccine availability. The COVID-19 Vaccines Global Access (COVAX), has officially approved Ukraine’s request for COVID-19 vaccines, recently estimated numbers of doses are 8 million. COVAX is co-led by Global Alliance for Vaccines and Immunisation (GAVI), the Coalition for Epidemic Preparedness Innovations (CEPI), and WHO. There is a plan to deploy 2 platforms: primary health-care and mobile teams. The Health Cluster plays an important role in bringing actors to the table to encourage regional coordination during pandemic disaster response. Immunization will be high on agenda, and not only for COVID-19, but also routine vaccination for vulnerable and at-risk communities.

## Medical Readiness: War and Pandemic Disease

In addition to treating the surge of patients with COVID-19, health-care facilities must continue to treat routine communicable and noncommunicable diseases. The medical readiness system in Ukraine will need to create reserves of medical equipment, medicines, medical devices, PPE, disinfectants, and capacity building and training. The global supply chain of medical supplies is as crucial to fighting COVID-19 as Ukraine’s military supply chain is crucial to fighting its military conflicts in defense of its sovereign borders. In response to these needs, the presidential directive is comprehensive and should offer great support and leadership to prepare Ukraine for the coming onslaught of COVID-19 cases.

Structurally, this new presidential direction and law will also focus on the restoration of the system of anti-epidemic protection, in particular the appointment of the main state public health doctors of the respective administrative-territorial units and approval of the procedure of temporary self-isolation of persons suspected of having COVID-19 disease, in an effort to decrease morbidity and mortality by decreasing the spread and incidence of coronavirus.

Due to the warfighting experience by Ukraine against unconventional and hybrid threats, experience was gained of plasma transfusion when fresh whole blood was not available for traumatic injuries. Transfusion of plasma with antibodies thus far has potentially promising results.^9^ Ukraine can serve as a testing ground for further studies based on the experience with plasma transfusion from war. The Ministry of Health of Ukraine will also be funded to purchase supportive medicines, medical equipment, and medical devices and supplies, including PPE, to fight COVID-19.

## Allocation of Scarce Resources

As in all countries facing this pandemic, allocation of resources, beds, and medical staffing requires strict prioritization. However, states at war and in conflict face significant challenges, as many efforts are already directed toward the line of contact and a formidable adversary. The situation demands immediate revision and optimization of plans for re-profiling of beds in health-care facilities belonging to the sphere of management of the Ministry of Health, and other institutions supporting health, and increasing the number of medical personnel and resources as COVID-19 cases rise. This includes ensuring the strict observance of fiscal discipline and the timely execution of payment orders during the procurement of goods, works, and services related to counteraction of COVID-19. Information and awareness-raising work to prevent COVID-19 and prevent it from spreading must be intensified as a policy, such as social distancing and personal hygiene, as does information sharing of data and needs assessments with international partners.

## Warfighting

Russia has not held any punches with the onset of COVID-19 as positive cases begin to spike. Since the first cases in late February 2020, there have been continued, daily, multiple ceasefire violations throughout the JFO by Russia and Russian proxies directly in defiance of the Minsk Agreements. Since January 2020, over 60 people have been killed. In violation of international law, Russia deploys prohibited weapon systems throughout Donbas, including 82-mm and 120-mm mortars and various multi-barrel rocket launchers (MBRLs). Throughout March and April 2021, Russia deployed over 100,000 troops along Ukraine’s entire eastern border and significantly increased its Black Sea presence.

Militarily, such provocations by Russia make sense: attack when your enemy is weak, whether by pandemic disease or otherwise. Will Russian aggression subside if morbidity and mortality among its own fighting force increases from COVID-19? Or will incursion increase and tactical advantage be capitalized upon? The persistent threat of Multi Domain Battle (MDB)/Multi Domain Warfare (MDW) effectiveness from Russia and Russian proxies throughout Donbas, Crimea, and the rest of Ukraine is severe. The threat will only increase as incidence and prevalence of COVID-19 remain a challenge for a fractured health-care infrastructure.

Ukraine Medical Forces received 100,000 rapid tests for COVID-19, and many troops have self-isolated, many have also already died. All military medical clinical centers, garrisons, and currently deployed mobile hospitals are receiving support to help prevent and treat COVID-19.

As of March 21, the Ukrainian Armed Forces are engaged in assisting units of the National Guard and Police, providing additional law enforcement on the streets of cities during the quarantine period announced by the Ministry of Interior. As of March 24, all Military Medical Departments will focus only on emergency treatment, because all elective operations have been postponed. Currently, the functional capacity is estimated at just over 50% across the military medical network. A lot of medical blocks were adopted for infectious diseases needs and observation, with over 500 beds added.

## Sanction Against the Russian Federation During the Pandemic

The leadership of the Russian Federation is taking consistent steps to strain and destroy NATO and EU states with a view to lifting international sanctions and eliminating Russian aggression in Donetsk and Luhansk and occupying the Crimean peninsula. On March 26, 2020, President Vladimir Putin proposed a moratorium on economic sanctions for countries affected by the epidemic. Such a proposal was criticized and found no support. Deputies of the European Parliament appealed to the EU leadership to leave sanctions in place. The deputies stressed the need for a ceasefire in Donbass, ensuring access of Ukrainian doctors and WHO representatives to the temporarily occupied territories to counteract COVID-19; in addition, Russian hybrid misinformation campaigns aim at discrediting the EU and erode democratic institutions and health security in general.^10^

## Institutions That Support Health Security

Within the immediate and disaster relief policy, Ukraine’s Ministry of Health established a closer working relationship with the Ministry of Foreign Affairs with the aim of facilitating receipt of humanitarian aid and procuring the necessary means and equipment to prevent and combat COVID-19. The Ministry of Health has also identified and will provide the predicted need for additional test systems to detect COVID-19 and ensure the continued availability of such systems in health=care institutions. The clinical algorithm on when and who to test is still under review. This requires health-care standards constantly being updated to best practices, to be in line with new COVID-19 therapy data from the WHO. Currently, infectious disease wards in Kyiv are strictly following the WHO guidelines for COVID-19 and are updated daily. Information sharing with partners will help the global community to learn from the Ukrainian COVID-19 experience.

By the order of the Minister of Health of Ukraine Number 772 (March 13, 2020), the immediate disaster response plan included the establishment and approval of the composition of operational headquarters to help prevent and mitigate new cases and further transmission of COVID-19 cases. This act also promotes best practice Health Care Standards and approval of COVID-19 Pharmaceutical Assistance Standards, to be updated regularly. Of note, as it relates to the controversial topic of testing, this new mandate approves the recommendations on actions of state institutions of regional and Kyiv city laboratories (including all modes of transport), centers of the Ministry of Health of Ukraine upon identification of the person who meets the COVID-19 case definition as defined and updated daily by the WHO. Many more resolutions and legal frameworks have been passed in support of health and health security with support from the Center for Public Health in the Ministry of Health for Ukraine.

Most notably, Dr. Igor Kuzin has been put in charge of the Center for Public Health seated in the Ministry of Health of Ukraine. This task force must provide an accurate and up-to-date list of countries with COVID-19 local transmission in accordance with WHO situational reports on its website and support with data analysis and epidemiological support for decision makers. Multiple hospitals have been identified in the city of Kyiv which can carry out tests for COVID-19, and in the whole of Ukraine, in each region, multiple medical institutions are involved in counteracting the pandemic through testing and direct treatment. The requirement of information sharing and integration of best practices across these facilities, and with international partners is urgently needed.

Additionally, the State Service of Ukraine for Food Safety and Consumer Protection, together with local executive authorities and with the participation of local self-government bodies, have urgently strengthened control over the water supply and sewerage systems. This policy feature in support of basic hygiene measures, is impossible in the Donbas for occupied regions and poses a threat to health for those populations; the central Government of Ukraine has no clinical or *de facto* governance for these regions.

Furthermore, the Ministry of Economy, Trade, and Agriculture of Ukraine urgently reviewed the nomenclature of the state reserve’s value and taxation systems, providing for a minimum supply of symptomatic treatment for acute respiratory illnesses and personal hygiene products, for a period of 3 months. The Ministry of Internal Affairs of Ukraine has ensured law enforcement while implementing measures to prevent proliferation COVID-19 and maintain rule of law. This includes recommendations that Ukrainian citizens refrain from traveling abroad to countries where COVID-19 cases are confirmed; additional travel and movement restrictions, international and nationals, are likely to evolve as the pandemic spreads. Increased testing services have been made available at ports of entry, and smartphone applications in support of self-isolation have also been deployed for citizens and visitors alike arriving in Ukraine from aboard.

## NATO and Ukraine

Ukraine has made its intentions to become a NATO full member state very clear. Relations between NATO and Ukraine date back to the early 1990s and have since developed into one of the most substantial of NATO’s partnerships. Since 2014, in the wake of the Russia-Ukraine conflict, cooperation has intensified in critical areas.^11^ Now, more than ever, is the time to cooperate, expand, integrate, and bolster NATO members and partners, by aligning their common interests against threats to their health security and shoring up combined disaster response efforts. Ukraine defense, health, and democratic governance structures must become more interoperable with NATO structures. NATO’s measures in support of Ukraine became part of the Comprehensive Assistance Package (CAP), which support Ukraine’s ability to provide for its own security and to implement wide-ranging reforms, as set out in Ukraine’s 2016 Strategic Defence Bulletin and NATO Trust Funds set up exclusively for Ukraine in support of health security and the defence sector.^12^

Some NATO members may close borders or restrict movement, cancel training engagements with neighboring partners, and focus on domestic security. This shortsighted approach will open the floodgates to increased health security threats, attack, and most importantly reduce the core objectives of the alliance: deter, contain, respond, and remain resilient to the violent, disruptive, or military efforts of others.^13^ Interoperability of NATO, NATO states, and partners and Ukraine must be increased and expanded upon across all domains of battle and disaster response. Another important objective is to develop operational capabilities and interoperability with NATO forces through a wide range of activities and military-to-military (M2M) exercises; this includes the Vigorous Warrior Live exercise series focused on damage control surgery and resuscitation (DCS/DCR) from point of injury (POI) to surgical intervention. Ukraine’s input on the DCR/DCS at this exercise and the NATO military medical center of excellence are vital to NATO troop readiness and interoperability for disaster response.

The threat of possible further intensified war with Russia calls for increased spending by European states and integration with new NATO members, such as Georgia and Montenegro, as well as key NATO partner nations, such as Ukraine, Moldova, Serbia, Finland, and Belarus. NATO deployed multinational Extended Forward Presence (EFP) missions to Latvia, Lithuania, and Estonia in 2016 in an effort to show solidarity with those states that are directly related to the Russian threat. The Alliance must not only show lockstep unity but also that the hand of friendship is strong and unaltering. There is no indication that NATO will call COVID-19 a challenge to the alliance, necessitating joint and effective action on the part of all the allies to craft a collective response, although this is possible. But most importantly, the instability and vulnerability of NATO and NATO members is at its highest point since the initiation of the alliance. Land, sea, air, space, and cyber domains are vulnerable to attack by an opportunistic adversary or sub-threshold Article 5 event.

NATO Secretary General Jens Stoltenberg presented his Annual Report for 2019 and outlined the Alliance’s response to the COVID-19 pandemic in mid-March 2020.

He emphasized the Article 5 defense, stating “Our forces remain ready and our work goes on, including in our multinational battlegroups in the east of the Alliance, NATO Air Policing, our maritime deployments, and our missions from Afghanistan to Kosovo”.^14^ Ukraine is in a unique position of not only being the only EU state in open and active combat with Russia and Russian backed proxies, but also dealing with the COVID-19 threat at the same time.

NATO activities that must be expanded and encourage 2-way information flow and sharing between Ukraine across the alliance include Knowledge Sharing and Situational Awareness (SA). NATO offers subject-matter expertise, documentation, training, standards, best practices, mentoring, and advice to Command, Control, Communications, Computers (C4) Intelligence, Surveillance, and Reconnaissance (ISR) (C4ISR) project teams, and subject-matter experts (SME) in Ukraine. SA provides a reference for the implementation of NATO standards, software SA tools such as Joint Operations Centre Watch (JOCWatch), the Secure Tactical Joint Chat Application Service (JChat), Interim Geo-Spatial Intelligence Tool, (iGeoSit), the EpiNATO-2 platform, the only interoperable health surveillance system and the to be released NATO Trauma Registry, procedures as well as support through mentoring and SME for Ukrainian capability development.

These efforts and many other NATO activities must be expanded upon and invested in and also feed into the NATO military medical center of excellence and the Lessons Learned (LL) process. Indeed, NATO has a lot to learn from the Ukrainian experience in fighting hybrid and multi-domain battle against a peer adversary. C4ISR challenges that Ukraine has overcome must be shared across the alliance to reduce and mitigate morbidity and mortality.

## CDC

CDC’s global health security efforts in Ukraine help improve the country’s ability to prevent, detect, and respond to infectious disease outbreaks before they become epidemics, as well as offer CDC key insight and data flow of Ukraine’s public health sector. Targets outlined in the Global Health Security Agenda (GHSA), a global partnership launched in 2014 to help make the world safer and more secure from infectious disease threats, are a core focus CDC, 2019.^15^

With CDC’s assistance, Ukraine has developed detailed action plans on disease surveillance, laboratory systems, workforce development, biosafety and biosecurity, immunization, and other critical areas.^15^ The CDC supports Ukraine in strengthening the capacity of its workforce to investigate and respond to disease outbreaks through the establishment of a Field Epidemiology Training Program (FETP); which trains field epidemiologists in promotion of evidence-based practices.

Every effort possible must expand this partnership through efforts that facilitate the implementation of these action plans to achieve the targets and competencies specified by GHSA and the WHO, bearing in mind that Ukraine faces the unique threat of being actively engaged in combat and war with Russia while it faces the challenges from COVID-19 pandemic. Last, the United States should share its pandemic response plan with Ukraine and train on interoperability. The CDC in Kyiv can act as a conduit to provide as much guidance and support as possible for the Ukrainian response to COVID-19, as well as learn from the new Ukrainian policy being implemented as this crisis unfolds.

## Defense Threat Reduction Agency

The US Defense Threat Reduction Agency (DTRA) enables the United States and partner nations “to counter and deter Weapons of Mass Destruction and Improvised Threat Networks”.^16^ DTRA has a history of working closely with Ukraine, including on the Cooperative Biological Engagement Program (BCEP), an effort focused on biosafety and biosecurity and biosurveillance lines of effort for the US geographic combatant commands. We encourage DTRA to step up capacity building efforts to train and supply Ukrainian epidemiologists and laboratories to handle the diagnostic and biostatistics requirements for responding to any infectious disease outbreak and integration biosurveillance, antimicrobial resistance, and chemical, biological, explosive, and nuclear (CBRN) threats. This must also include expanded interagency cooperation and support of epidemiological data sharing and exchanges across scientific disciplines in support of health security structures.

## Discussion

Ukraine and its partners for security must leverage advancements to support international cooperative programs that aim at lowering biological and health security threats and build scientific capacity, which in turn may accelerate stability for a country at war. NATO members should continue to share preparedness plans and disaster response best practices as evidence-based policy focused on COVID-19 are discovered. To get through the pandemic together, states must learn from one another and rapidly implement any containment, mitigation, treatment, and rapid vaccine rollout options.

## Conclusions

Ukraine faces significant challenges to its health security and state sovereignty. The COVID-19 novel coronavirus pandemic exacerbates open fissures in the democratic institutions for health, security, and governance. Now is the time to increase integration efforts across domains to secure Ukraine’s ability to fight the aggressor Russia, provide health security for its citizens, and be an active member of the international community’s fight against the pandemic. Increased support for a NATO member would conform to the security alliance’s support of democratic institutions and state sovereignty. NATO offers an anchor of support for these efforts through open and transparent partnership. Ukraine is a young and fragile state, with the potential to offer massive contributions to the social, economic, political, academic, and scientific community across Europe and beyond. The opportunity must not be missed to further integrate and indoctrinate Ukraine and its institutions into a broader and more transparent alliance.

## Footnotes

a This rate rose to 2% in mid spring 2021, where nearly 2 million people were infected with the coronavirus disease 2019 (COVID-19), over 40,000 dead.
